# Serum neopterin levels in relation to mild and severe COVID-19

**DOI:** 10.1186/s12879-020-05671-7

**Published:** 2020-12-10

**Authors:** Josefina Robertson, Johanna M. Gostner, Staffan Nilsson, Lars-Magnus Andersson, Dietmar Fuchs, Magnus Gisslen

**Affiliations:** 1 Department of Infectious Diseases, Institute of Biomedicine, Sahlgrenska Academy, University of Gothenburg, Gothenburg, Sweden; 2 Department of Infectious Diseases, Region Västra Götaland, Sahlgrenska University Hospital, Gothenburg, Sweden; 3 Institute of Medical Biochemistry, Biocenter, Medical University of Innsbruck, Innsbruck, Austria; 4 Mathematical Sciences, Chalmers University of Technology, Gothenburg, Sweden; 5 Institute of Biological Chemistry, Biocenter, Medical University of Innsbruck, Innsbruck, Austria

**Keywords:** COVID-19, Prognostic markers, Neopterin, Tryptophan, Kynurenine

## Abstract

**Background:**

The COVID-19 pandemic, caused by the coronavirus SARS-CoV-2, is rapidly spreading worldwide. There is limited information about prognostic markers that could help clinicians to identify COVID-19 patients with a poor prognosis. Serum levels of the immune activation marker neopterin has shown to be of prognostic value in patients with SARS. The aim of this study was to investigate whether serum neopterin is associated with the severity of COVID-19.

**Methods:**

We included 34 patients with confirmed COVID-19 between March 3 and March 30, 2020. Fifteen patients had mild disease and did not require hospitalization, whereas 19 patients developed severe COVID-19 requiring intensive care. Concentrations of serum neopterin, tryptophan, and kynurenine were measured at and repeatedly after inclusion.

**Results:**

We found a more than two-fold higher mean concentration of neopterin in severely ill patients (mean value 42.0 nmol/L (SD 18.2)) compared to patients with mild symptoms (16.9 nmol/L (SD 11.0)). All of the severe cases had elevated neopterin concentrations (> 9.1 nmol/L) at the initial sampling with values ranging from 17.2 to 86.7 nmol/L. In comparison, 10 of 15 patients with mild disease had neopterin levels above 9.1 nmol/L, with concentrations in the range from 4.9 to 31.6 nmol/L. Neopterin levels gradually decreased during the course of COVID-19, but severe cases maintained elevated levels for a longer period. Moreover, lower levels of tryptophan and higher levels of kynurenine, indicating an increased tryptophan catabolism, were seen in the group with severe cases.

**Conclusions:**

In conclusion, we found that serum neopterin levels are associated with the severity of COVID-19. Our findings suggest that neopterin could be used as a prognostic marker, but further studies are needed to elucidate how it can be used in the clinic.

## Introduction

The COVID-19 pandemic, caused by the coronavirus SARS-CoV-2, is rapidly spreading worldwide [1]. Most patients have mild symptoms from the upper respiratory tract, whereas a minor but not negligible proportion suffers from a severe form of the disease, which in some cases require intensive care [2]. There is limited information about prognostic markers that could help clinicians to identify COVID-19 patients with a poor prognosis. During the outbreak of the severe acute respiratory syndrome (SARS) in 2002–2003, caused by the similar coronavirus SARS-CoV, levels of the immune activation marker neopterin were found to predict the course of disease [3].

Neopterin (6-(D-erythro-1′, 2′, 3′-trihydroxypropyl)-pterin) is a well-established immune activation marker with elevated concentrations seen in many inflammatory states including infections, autoimmune disorders, and cancer [4]. In acute viral infections such as hepatitis [5], Cytomegalovirus disease [6], Rubella [7], and dengue fever [8], serum neopterin levels correlate with the activity of the disease, and can be detected before antibody production [4]. The elevation of neopterin originates mainly from the increased synthesis by human monocyte-derived macrophages, whereby interferon-gamma (IFN-y) is the most central activating cytokine [9]. IFN-y also promotes the conversion of the essential amino acid L-tryptophan (TRP) to N-formylkynurenine, which is rapidly converted into the more stable kynurenine (KYN), by induction of the anti-proliferative and immunoregulatory enzyme indoleamine 2,3-dioxygenase (IDO). The first step of the TRP breakdown is the rate-limiting step in the TRP catabolic route along the KYN axis, and the KYN to TRP ratio can be used as a measure of the IDO enzyme activity [10]. Taken together, IFN-y mediated immune response to viral infections may lead to elevated neopterin levels, as well as increased TRP degradation and elevated KYN to TRP ratio [4, 11].

In patients with SARS, elevated levels of neopterin were detected already at the day of symptom onset, and rose to a maximum level at day 3 [3]. Moreover, patients with higher levels of neopterin at an early stage suffered from a severer course of disease, including higher and longer period of fever, more severe dyspnea, longer hospitalization, and more complications [3]. Considering these findings and the similarity of the coronaviruses, neopterin may be a useful prognostic marker for the course of COVID-19. The aim of this study was to investigate whether serum neopterin levels in COVID-19 patients are associated with the severity of disease.

## Methods

### Participants

We included 34 patients with COVID-19, who were admitted to the Department of Infectious Diseases at the Sahlgrenska University Hospital, Gothenburg, Sweden. All cases were confirmed with reverse transcriptase polymerase chain reaction (RT-PCR) from nasopharyngeal and throat aspirates. Fifteen patients had mild disease and did not require hospitalization, whereas 19 patients developed severe COVID-19 that in most cases required intensive care. Blood samples were collected between March 3 and March 30, 2020.

### Serum neopterin, TRP, KYN, and IFN-y measurements

Serum neopterin concentrations were determined using enzyme-linked immunosorbent assay (ELISA) (BRAHMS GmbH, Hennigsdorf, Germany) as described by the manufacturer’s instructions. Serum samples and standards were treated with Igepal (Sigma-Aldrich, Vienna, Austria; final concentration in serum or standards was 2% (v/v)). Sensitivity of the test was 2 nmol/L neopterin. The upper normal reference level was 9.1 nmol/L in serum [12]. Serum TRP and KYN concentrations were measured by a reverse-phase HPLC method [13], using a Varian ProStar HPLC system equipped with a solvent delivery module (model 210), an autosampler (model 400, both Varian ProStar), an UV-spectrometric detector (SPD-6A, Shimadzu), and a fluorescence detector (model 360, Varian ProStar). Varian Star Chromatography Workstation (version 6.30) software was used. To analyze serum IFN-y, we used the human IFN-gamma Quantikine ELISA (R&D-DIF50C) according to the manufacturer’s instructions.

### Statistical analyses

Descriptive statistics are shown for all variables involved in the analyses, presented as means with standard deviations. For statistical analyses, continuous variables were log_10_ transformed. Student’s t-test was used for group comparisons. Changes in log concentrations from first to last measure were analyzed with paired t-test. Associations were measured with Pearson correlation. To study the change in neopterin levels over time, we used a linear mixed effects model with severity (mild/severe), days since symptom onset, and age as covariates. An interaction term between severity and days since onset was included in the model. All statistical analyses were performed with the Statistical Package for the Social Sciences (SPSS) software version 25 (SPSS, Chicago, Illinois, USA) or Prism (GraphPad software version 8.0, La Jolla, California, USA). A significance level below 0.05 was considered as statistically significant.

## Results

The study population comprised 34 patients with COVID-19, of which 15 displayed mild symptoms (mean age 51.3 years (SD 13.7)), and 19 developed a severe form of the disease (mean age 61.3 years (SD 11.4)). Cough, myalgia, nasal congestion, fever, and sore throat were the most common symptoms among mild cases, whereas fever, cough, dyspnea, and fatigue were most frequent in severe cases (Table 1). Among severe cases, all but four required invasive mechanical ventilation, and three were in need of continuous renal replacement therapy. Four deaths occurred in this group. Comorbidities among mild cases were rare, while a majority of severe cases had one or more comorbidities, of which hypertension and diabetes mellitus were most frequent (Table 1).

**Table 1:** Clinical characteristics of included patients with mild and severe COVID-19

	Mild disease (*n* = 15)	Severe disease (*n* = 19)
Gender (M/F)	6/9	17/2
Age, years (SD)	51.3 (13.7)	61.3 (11.4)
Comorbidity, n (%)	2 (13)	11 (58)
Hypertension	0 (0)	11 (58)
Diabetes mellitus	1 (7)	6 (32)
Coronary heart disease	1 (7)	3 (16)
Chronic obstructive pulmonary disease	0 (0)	1 (5)
Asthma	0 (0)	1 (5)
Symptoms, n (%)		
Fever	9 (60)	19 (100)
Cough	14 (93)	18 (95)
Dyspnea	1 (7)	17 (89)
Fatigue	3 (20)	12 (63)
Myalgia	11 (73)	5 (26)
Sore throat	8 (53)	2 (11)
Nasal congestion	11 (73)	1 (5)
Intensive care, n (%)	0 (0)	17 (89)
Invasive mechanical ventilation, n (%)	0 (0)	15 (79)
Continuous renal replacement therapy, n (%)	0 (0)	3 (16)
Death, n (%)	0 (0)	4 (21)

*M* males, *F* females

**Fig. 1:**
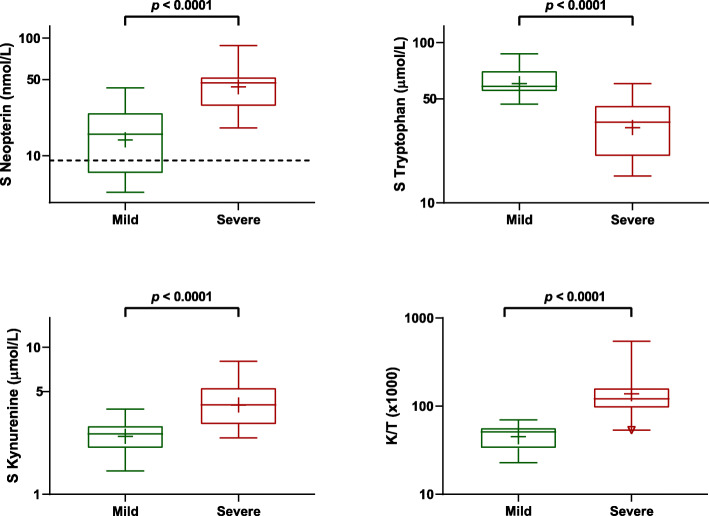
Concentrations of first measurements of serum neopterin, tryptophan, and kynurenine, as well as kynurenine to tryptophan ratio (K/T) in patients with mild (green) and severe (red) form of COVID-19 (*n* = 34). The dashed line represents the upper normal reference limit of neopterin at 9.1 nmol/L.

**Fig. 2:**
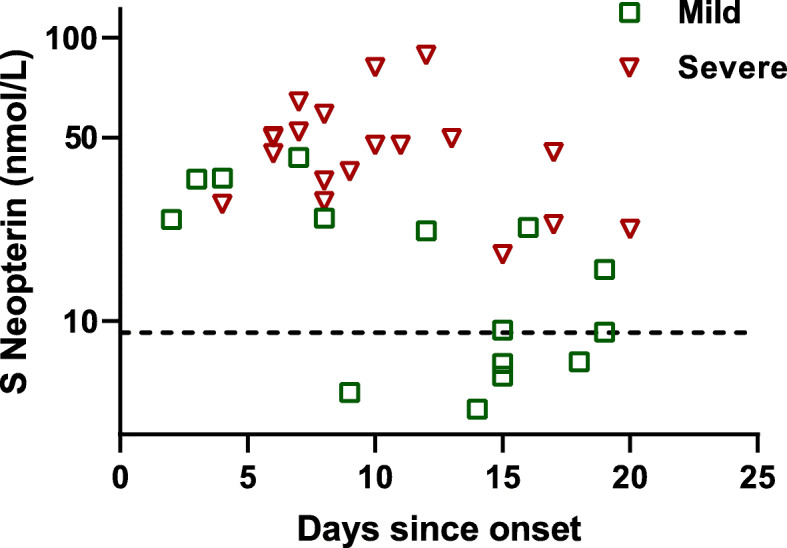
First measurements of serum neopterin concentrations in patients with mild (green) and severe (red) form of COVID-19 (*n* = 34). The dashed line represents the upper normal reference limit of neopterin at 9.1 nmol/L.

To test whether neopterin levels are associated with the severity of COVID-19, we compared serum neopterin concentrations between patients with mild and severe disease. We found a more than two-fold higher mean concentration of neopterin in severely ill patients (mean value 42.0 nmol/L (SD 18.2)) compared to patients with mild symptoms (16.9 nmol/L (SD 11.0)) (Table 2, Figs. 1 and 2). All of the severe cases had elevated neopterin concentrations (> 9.1 nmol/L) at the initial sampling with values ranging from 17.2 to 86.7 nmol/L, measured at day 4–20 (mean 11.7 (SD 5.8); median 14) since onset of symptoms. In comparison, 10 of 15 patients with mild disease had neopterin levels above 9.1 nmol/L at day 2–19 (mean 10.2 (SD 9); median 9), with concentrations for the entire group in the range from 4.9 to 31.6 nmol/L. These results show that patients with severe COVID-19 display higher levels of neopterin than mild cases. Since renal insufficiency may affect serum neopterin levels [14], we analyzed creatinine levels in relation to neopterin in severe cases. However, no significant correlation was found (*r =* 0.36, *p* = 0.13). Moreover, we found no association between neopterin levels and C-reactive protein (CRP) or B-lymphocytes among the severely ill (data not shown).

**Table 2:** Concentrations of neopterin and amino acids from the study population divided into two groups based on disease severity

	Mild (*n =* 15) Mean (SD)	Severe (*n =* 19) Mean (SD)	*p*-value
Days since onset	10.2 (9.0)	11.7 (5.8)	
Neopterin, nmol/L	16.9 (11.0)	42.0 (18.2)	*p <* 0.0001
Tryptophan, μmol/L	56.3 (11.6)	31.9 (13.0)	*p <* 0.0001
Kynurenine, μmol/L	2.6 (0.7)	4.3 (1.4)	*p <* 0.0001
KYN/TRP *1000	47.5 (15.1)	163.4 (117.5)	*p <* 0.0001
IFN-y, pg/mL	6.7 (5.0)	21.4 (20.8)	*p <* 0.001

*Means and standard deviations (SD) are shown*

*KYN/TRP* kynurenine to tryptophan ratio, *IFN-y* interferon-gamma

**Fig. 3:**
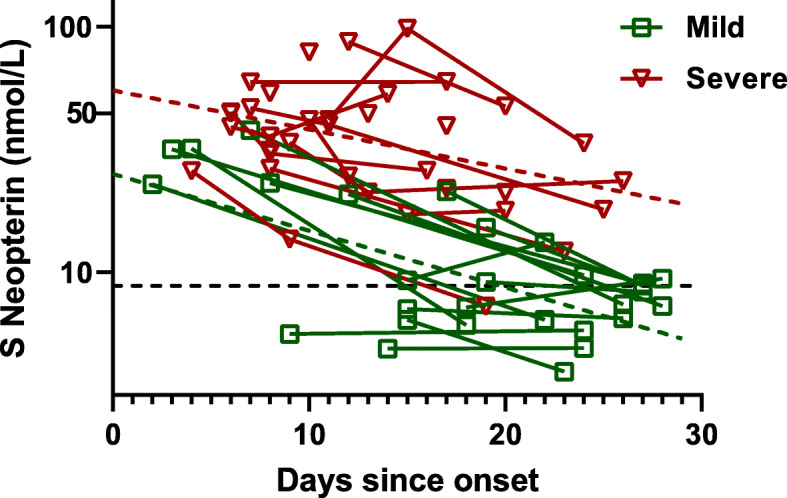
Repeated measurements of serum neopterin concentrations in patients with mild (green) and severe (red) form of COVID-19 (*n* = 34). Severity (mild/severe), days since symptom onset, and age were used as covariates in the linear mixed effect model. The black dashed line represents the upper normal reference limit of neopterin at 9.1 nmol/L.

As a next step, we studied the trajectory of neopterin during the progression of COVID-19 by repeated measurements. We found that serum neopterin levels decreased over time, regardless of mild or severe disease (*p* < 0.0001, Fig. 3). At day 14 after symptom onset, the group with severe disease displayed 2.3 times higher neopterin levels than patients with mild disease. Moreover, neopterin concentrations decreased 23% per week in severe cases compared to 31% per week in mild cases. The difference in slope was, however, not significant (*p* = 0.23). Twelve of the patients with mild disease had normal levels (< 9.1 nmol/L) at the last measurement. In contrast, only one patient among the severely ill returned to a normal level during the study period (Fig. 3). These results show that neopterin levels gradually decrease during the course of COVID-19, but that severe cases maintain elevated levels for a longer period. Collectively, our findings indicate an association between neopterin and the severity of COVID-19.

**Fig. 4:**
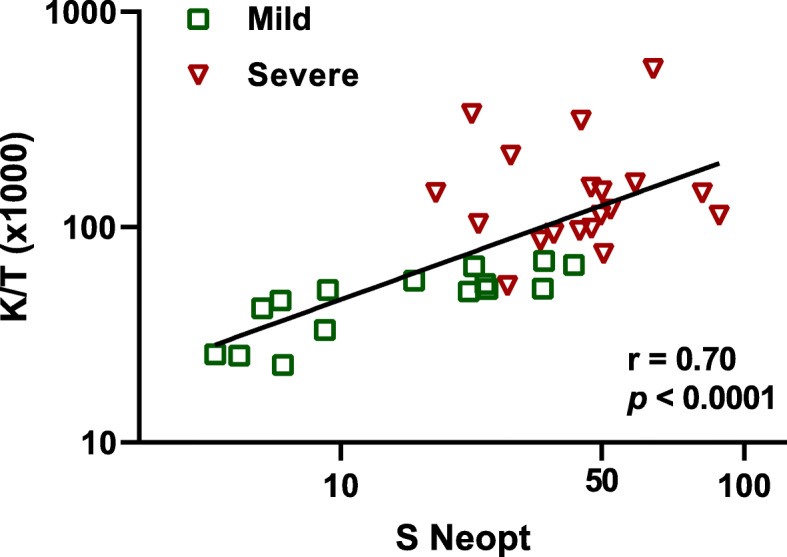
The correlation between first measurements of serum neopterin (S Neopt) concentration and kynurenine to tryptophan ratio (K/T) in patients suffering from COVID-19 (*n* = 34)

Furthermore, we investigated TRP, KYN, and IFN-y concentrations in relation to mild and severe COVID-19. As seen in Fig. 1, we found lower levels of TRP and higher levels of KYN in severe cases, as compared to mild cases. This indicates that a severe form of COVID-19 is associated with an increased TRP catabolism. Additionally, KYN/TRP concentrations closely correlated to neopterin (*r =* 0.7, *p* < 0.0001, Fig. 4). Analysis of IFN-y in serum revealed significantly higher levels in severe cases compared to mild cases (Table 2), and the levels of IFN-y correlated to neopterin (*r =* 0.8, *p <* 0.0001).

## Discussion

In the present study, we investigated if serum neopterin levels in patients with COVID-19 are associated with the severity of disease. We report that higher neopterin concentrations were seen in patients who developed severe disease compared to patients with mild disease. In the group with severe COVID-19, we also found an increased metabolism of TRP, as expressed by higher KYN to TRP ratio.

The observed difference in neopterin concentrations between mild and severe COVID-19 is in agreement with previous results from SARS patients [3]. In that study, higher neopterin levels were associated with a longer fever period, as well as a severer course of disease. Moreover, a recent report on hospitalized COVID-19 patients showed that neopterin levels were significantly higher in severe cases compared to mild cases [15]. Besides supporting these findings, our study adds the trajectory of neopterin levels with repeated measurements, where we found that most patients with mild symptoms returned to normal neopterin levels at the end of the study period. In contrast, neopterin levels remained elevated during the course of disease in the severely ill. This probably illustrates a more advanced and prolonged inflammatory state, as suggested by others [16].

The elevated levels of neopterin and IFN-y in severely ill patients with COVID-19 indicate a potent stimulation of the monocyte/macrophage–T cell interplay during the symptomatic period [9]. Our detection of high neopterin concentrations already at day 2 after symptom onset suggests an early inflammatory response to SARS-CoV-2. Likewise, the study of SARS patients [3], and also a study of dengue fever [8], found neopterin elevation at the first day of symptoms. The characteristics of neopterin being elevated at an early stage of disease, as well as being associated to disease severity, suggest it as a useful prognostic marker for COVID-19.

The increased KYN to TRP ratio in patients with severe COVID-19 reflects an increased TRP catabolism. Similar findings have been presented for HIV-infected patients with a progressive disease [17]. TRP deprivation is an effective strategy of the Th1-type immune response to reduce undesirable proliferation of pathogens and infected cells [18], which may be useful for disease control in COVID-19.

When interpreting our results, one must consider the potential involvement of renal function [14]. Acute kidney injury has been found in 50% of fatal COVID-19 cases [19]. In this context, the lack of creatinine measurements in mild cases make out a limitation of the present study. However, we found no correlation between creatinine and neopterin levels in the severe cases at the first measurement.

## Conclusions

The present study shows an association between neopterin levels and severity of COVID-19, and also elevated concentrations early in disease progression. In conclusion, serum neopterin is a potential marker for the prognosis of COVID-19 when detected in blood samples from a few days since symptom onset, but further studies are needed to elucidate how it can be used in the clinic.

## Data Availability

The datasets used and/or analyzed during the current study are available from the corresponding author on reasonable request.

## Abbreviations

SARS: Severe acute respiratory syndrome
IFN-y: Interferon-gamma
TRP: Tryptophan
KYN: Kynurenine
IDO: Indoleamine 2,3-dioxygenase
RT-PCR: Reverse transcriptase polymerase chain reaction
ELISA: Enzyme-linked immunosorbent assay
CRP: C-reactive protein
